# Identification of Subclinical Myocardial Dysfunction in Breast Cancer Patients with Metabolic Syndrome after Cancer-Related Comprehensive Therapy

**DOI:** 10.1155/2021/6640673

**Published:** 2021-03-02

**Authors:** Feng Zhang, Siyuan Wang, Siying Liang, Chao Yu, Sufang Li, Hong Chen, Shu Wang, Tiangang Zhu

**Affiliations:** ^1^Department of Cardiology, Peking University People's Hospital, Beijing, China; ^2^Beijing Key Laboratory of Early Prediction and Intervention of Acute Myocardial Infarction, Peking University People's Hospital, Beijing, China; ^3^Department of Breast Surgery, Peking University People's Hospital, Beijing, China

## Abstract

**Background:**

Breast cancer patients with metabolic syndrome have an increased risk of cardiovascular disease. These patients are more prone to suffer from cardiotoxicity after anticancer therapy. Patients after completion of cancer-related comprehensive therapy, who show normal myocardial function, may already have subclinical myocardial dysfunction. We sought to evaluate the subclinical myocardial dysfunction in breast cancer patients with metabolic syndrome after cancer-related comprehensive therapy. Methods. In this study, 45 breast cancer patients with metabolic syndrome after completion of cancer-related comprehensive therapy, 45 non-breast cancer patients with metabolic syndrome, and 30 breast cancer patients without metabolic syndrome after therapy were enrolled. Left ventricular ejection fraction (LVEF) and global longitudinal strain (GLS) were measured using echocardiogram.

**Results:**

All the patients had normal LVEF. However, nine breast cancer patients with metabolic syndrome (20%) had GLS that was lower than –17%, while all the noncancer patients had normal GLS. Breast cancer patients with metabolic syndrome had a decrease of GLS and LVEF, compared with noncancer patients with metabolic syndrome. Furthermore, we found that decrease of age was associated with reduction of LVEF and that use of trastuzumab for 1 year was a significant factor associated with reduction of GLS. In addition, breast cancer patients with metabolic syndrome had a decrease of GLS, compared with breast cancer patients without metabolic syndrome after cancer-related therapy.

**Conclusions:**

Breast cancer patients with metabolic syndrome after completion of cancer-related comprehensive therapy suffered from subclinical myocardial dysfunction. GLS should be routinely performed to early identify subclinical myocardial damage of patients, in order to prevent the cardiotoxicity of cancer-related comprehensive therapy.

## 1. Introduction

The incidence of metabolic syndrome has increased year by year with the unhealthy lifestyle. Previous study has estimated that 24.5% of Chinese subjects over 15 years old had suffered from metabolic syndrome [1]. Recently, several studies have shown that metabolic syndrome is involved in the occurrence, recurrence, and metastasis of breast cancer, thus affecting the prognosis of breast cancer patients [2]. In China, there are a large number of breast cancer patients with metabolic syndrome, especially those with abdominal obesity. It is known that patients with metabolic syndrome have an increased risk of cardiovascular disease [3]. Furthermore, most of these breast cancer patients need to have cancer-related comprehensive therapy, including chemotherapy, targeted therapy, and radiotherapy, which may result in the further damage of their myocardium.

Recent studies have shown that chemotherapy, targeted therapy, and radiotherapy in breast cancer patients can cause injury of the myocardium. Use of anthracyclines, one of most widely used chemotherapeutic drugs, can lead to acute and chronic toxic damage to myocardium [4]. In addition, trastuzumab is often used in combination in HER2 or ErbB2 positive breast cancer patients. Although trastuzumab improves clinical outcomes by targeting the tumor, trastuzumab also causes an increased risk of cardiovascular adverse events, the most common of which is the left ventricular systolic dysfunction [5]. Furthermore, radiation therapy for left breast cancer can also cause cardiotoxicity, including cardiac insufficiency, due to its radiation to the heart [6].

The European Society of Cardiology have defined that cancer therapy-related cardiac dysfunction (CTRCD) is that the reduction of left ventricle ejection fraction (LVEF) is over 10% or LVEF is decreased to a value below 50% [7]. However, the decrease of LVEF that can be detected by echocardiogram may occur after sever damage of myocardium in patients. In more than half of these patients, left ventricular dysfunction has been permanently impaired and cannot be restored [8]. It was found that measurement of GLS using two-dimensional speckle tracing echocardiography (STE) can detect early change of left ventricular function, thus predicting the occurrence of CRTCD [9]. However, due to the insufficient understanding of GLS, it is not routinely used in cancer patients [10]. In this study, we aim to evaluate the cardiotoxicity of patients by both LVEF and GLS, together.

Rare studies focused on the cardiotoxicity of cancer-related therapy in patients with metabolic syndrome. Therefore, our study observed the cardiac function in breast cancer patients with metabolic syndrome, compared with that in noncancer patients with metabolic syndrome. We aimed to find the subclinical myocardial dysfunction of these patients and related risk factors, so as to early prevent cardiotoxicity in these patients.

## 2. Methods

### 2.1. Study Design and Population

In our study (http://www.chictr.org.cn Identifier: ChiCTR1900022108), 45 breast cancer patients with metabolic syndrome who were admitted to Breast Center of Peking University People's Hospital from November 2018 to February 2019 were consecutively enrolled. The inclusion criteria were as follows: (1) patients were ≥18 and ≤60 years old; (2) patients were diagnosed with stage I-III breast cancer; (3) patients have completed breast cancer surgery, chemotherapy, targeted therapy, and radiotherapy in Breast Center; (4) patients had a body weight change of less than 10% in the past 6 months; (5) patients had a waist circumference ≥80 cm with at least one abnormal indicator, including high blood glucose, high blood pressure, and dyslipidemia. The exclusion criteria included the following: (1) patients had heart-, liver-, or kidney-related diseases; (2) Patients had history of other caner. Age, height, weight, radiotherapy, targeted therapy and chemotherapy regimen, and cardiovascular risk factors were collected for each patient. Meanwhile, serum type B natriuretic peptide (BNP) and troponin I (TnI) were also collected. In addition, 45 noncancer patients with metabolic syndrome were enrolled as matches. The inclusion criteria were (1), (4), (5), that the same with breast cancer patients, and no history of cancer, as well as no heart, liver, and kidney related disease. Moreover, 30 breast cancer patients without metabolic syndrome were enrolled as well. The inclusion criteria were (1), (2), and (3), but without (5).

### 2.2. Image Acquisition

All patients were examined by transthoracic echocardiography and contrast-enhanced echocardiography using GE95. All echocardiographic exams were performed by the same technician using the same machine. All images were interpreted by the same cardiologist. Contrast-enhanced echocardiography for left ventricular opacification (LVO) was used to improve the accuracy of quantitative assessment of LVEF. LVEF was calculated by the two-plane Simpson method. GLS was measured in all patients. The specific measurement is as follows. When the images were collected, we made an optimization of the gain, compress, and time-gain compensation controls to get clear appearance of the left ventricle. Then, apical views (4, 2, and 3 chambers) were collected using high frame rate (>50 frames/s). The GLS was measured, and the boundary tracking was optimized by manual corrections. The images of each patient had no or just one segment of poor display.

All participants provided written informed consent. The study was approved by the Medical Ethics Committee of Peking University People's Hospital.

### 2.3. Statistical Analysis

Continuous variables were represented by mean ± standard deviation. Categorical variables were expressed by percentage of patients in each group. Categorical variables were compared using Person's chi-square test. Continuous variables were compared using independent sample *t*-test. Multiple linear regression was used to analyzed the risk factors related to GLS and LVEF. *P* < 0.05 was considered to have statistical significance. All the analyses were performed by SPSS 20.0 software.

## 3. Results

Baseline characteristics of the 45 breast cancer patients with metabolic syndrome and the 45 noncancer patients with metabolic syndrome are shown in Table 1. The mean age of breast cancer patients was 49 years. The mean BMI of breast cancer patients was 27.8 kg/m^2^. There were no significant differences in age, BMI, waist circumference, and cardiovascular risk factors between breast cancer patients and noncancer patients. All of the breast cancer patients with metabolic syndrome showed TnI and BNP levels within normal range.

**Figure 1:**
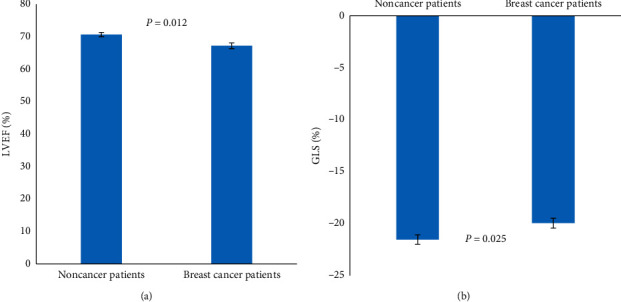
LVEF (a) and GLS (b) in breast cancer patients with metabolic syndrome and noncancer patients with metabolic syndrome.

All patients had normal LVEF. However, four breast cancer patients with metabolic syndrome had LVEF that was lower than 60%, while no noncancer patients with metabolic syndrome had LVEF that was lower than 60%. Furthermore, among the breast cancer patients with metabolic syndrome, nine patients (20%) had GLS that was lower than −17%, which is the normal lower limit of GLS. In contrast, there was no abnormality of GLS in the noncancer patients with metabolic syndrome. In addition, we found that the breast cancer patients with metabolic syndrome had a decrease of GLS and LVEF (GLS −19.95 ± 2.98%, LVEF 67.19 ± 5.92%), compared with the noncancer patients with metabolic syndrome (GLS −21.53 ± 2.32%, LVEF 70.63 ± 3.24%) (Figures 1(a) and 1(b)).

Multivariate linear regression analysis was performed to identify possible factors that affected LVEF and GLS in breast cancer patients. Age, BMI, use of anthracycline, use of trastuzumab, and left-side radiotherapy were included. We found that age was a significant factor that affected LVEF (Table 2). Specifically, decrease of age was associated with decrease of LVEF. Moreover, use of trastuzumab was a significant factor that was associated with reduction of GLS (Table 3).

**Figure 2:**
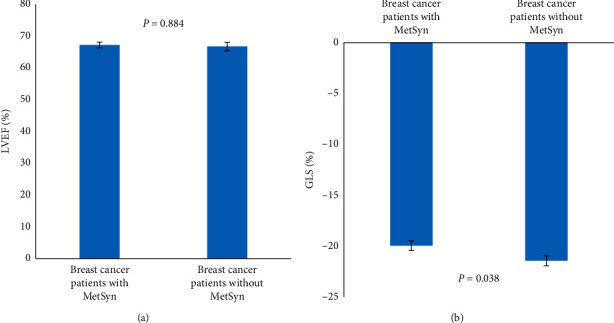
LVEF (a) and GLS (b) in breast cancer patients with metabolic syndrome (MetSyn) and breast cancer patients without metabolic syndrome (MetSyn) after cancer-related therapy.

In addition, we also enrolled the breast cancer patients without metabolic syndrome after cancer-related comprehensive therapy (Supplementary Table S1) and compared them with the breast cancer patients with metabolic syndrome. The mean BMI of the breast cancer patients without metabolic syndrome was 22.8 kg/m^2^, which was much lower than that of the breast cancer patients with metabolic syndrome (27.8 kg/m^2^). We found that there was no significant difference in LVEF between the two groups. However, GLS decreased in breast cancer patients with metabolic syndrome (GLS −19.95 ± 2.98%, LVEF 67.19 ± 5.92%), compared with breast cancer patients without metabolic syndrome (GLS −21.43 ± 2.73%, LVEF 66.69 ± 6.93%) (Figure 2). Only 3 of the breast cancer patients without metabolic syndrome (10%) had GLS <17%, and the proportion was lower than the that of breast cancer patients with metabolic syndrome (20%).

## 4. Discussion

In this study, we observed the subclinical myocardial dysfunction of breast cancer patients with metabolic syndrome after completion of cancer-related comprehensive therapy. We measured LVEF and GLS in these patients, compared those with noncancer patients with metabolic syndrome, and identified risk factors that may be associated with subclinical myocardial dysfunction.

Our main findings are as follows: (1) breast cancer patients with metabolic syndrome after completion of cancer-related comprehensive therapy have decreased LVEF and GLS, compared to those without cancer, even though their LVEF are all within normal range; (2) decreased age is the risk factor of LVEF reduction in breast cancer patients with metabolic syndrome, while use of trastuzumab is associated with the reduction of GLS.

Our study used both LVEF and GLS to observe the myocardial injury of breast cancer patients with metabolic syndrome. LVEF is the regular method to be used in evaluation of myocardial function in cancer patients with tumor-related therapy. In contrast, echocardiography-based myocardial strain is a novel way to detect subclinical dysfunction of left ventricle. GLS may be a more sensitive predictor of toxicity of heart, compared to LVEF. This may be explained by the following reasons. Chemotherapy may affect just certain segments of left ventricle, resulting in the early reduction of GLS. Other region of left ventricle may have compensatory enhanced movement, leading to unchanged LVEF [11]. In addition, LVEF may be affected by many other conditions including preload, heart rate, etc. [12]. Tracing process is often used in measurement of LVEF. In contrast, GLS may adopt more accurate measurement through STE (speckle tracking echocardiogram). Therefore, 2014 ASE/EACVI Expert Consensus recommend that GLS can be used to early detect subclinical dysfunction of left ventricle in the patients with chemotherapy [13]. Indeed, we observed that there was a reduction of GLS in the breast cancer patients with metabolic syndrome after treatment and that 9 breast cancer patients with normal EF, however, had GLS below normal lower limit, indicating that GLS can be effective in finding early subclinical myocardial dysfunction [14, 15].

In addition, the average time after the completion of cancer-related comprehensive therapy of the breast cancer patients in our study was 33 months. Although TnI, BNP, and LVEF of these patients were in the normal range, 20% of these patients had an abnormal GLS, suggesting that the subclinical myocardial injury may persist for a long time after completion of anticancer therapy. Therefore, GLS should be used to monitor the early myocardial injury over a long period of time even after completion of cancer-related comprehensive therapy.

In our study, we found that the reduction of age was associated with LVEF reduction. Previous studies have shown that age >65 years is a risk factor for cardiotoxicity of cancer therapy [16]. However, we found that LVEF reduction more easily occurred in younger patients. This may be due to the fact that the average age of the patients in our study was 49 years old, with the youngest being 34 years old. Similarly, some studies have shown that the incidence of cardiotoxicity was elevated in younger patients. It was also found in a study with patients younger than 41 years old that there was a 6-fold increased risk of death resulting from cardiovascular diseases in patients treated for Hodgkin's Disease before age 21, and that this elevated mortality decreased with increase of age [17]. The higher risks in patients treated at a younger age may be explained by a cardiovascular tissue more vulnerable to cancer-related therapy. In addition, the average age of patients treated with both anthracycline and trastuzumab in our study was 43 years, while the average age of the other patients was 51 years. This indicates that more younger patients received chemotherapy containing anthracycline plus targeted therapy in our study, while older patients chose noncombination therapy of anthracycline and trastuzumab. This may also explain the association of the reduction of age with LVEF reduction.

Our study found that trastuzumab can cause a decrease in GLS, which has been confirmed by many other studies. The mechanism of GLS reduction induced by trastuzumab is that it binds to HER-2 receptor of myocardial cells, which leads to the imbalance of Bcl-xL and Bcl-sL and sequential activation of mitochondrial apoptotic pathway. These result in myocardial injury and the decrease of GLS. Myocardial injury induced by trastuzumab, which is classified as type II CTRCD, leads to indirect cell injury and may be partly recovered after withdrawal of trastuzumab [5]. We do not find a relationship between use of anthracycline and cardiotoxicity. This may be explained by the fact that the cumulative dosage of anthracycline for all patients that receive anthracycline therapy in our study is 400 mg/m^2^, which is below the waning dose of cardiotoxicity [18]. In addition, the number of patients in our study is small, and large-scale studies are still needed to further verify.

In our study, we did not find the effect of left-side radiotherapy on the LVEF and GLS, which may be related to the use of intensity-modulated radiation therapy (IMRT) in our breast center. IMRT is a new radiotherapy technology that can reduce adverse events of radiotherapy [19]. IMRT allows for the radiation dose to conform more precisely to the three-dimensional (3D) shape of the breast cancer by modulating the intensity of the radiation bean in multiple small volumes. IMRT also allows higher radiation doses to focus on the tumor while minimizing the dose to surrounding normal critical structures, including heart. Previous studies have shown that IMRT could effectively reduce clinical toxicities compared with conventional breast radiotherapy [20, 21].

We included patients with metabolic syndrome in our study and found that breast cancer comprehensive therapy caused subclinical myocardial dysfunction, compared with those without anticancer therapy. The metabolic syndrome has become a worldwide problem. The metabolic syndrome includes abdominal obesity, hyperlipidemia, hypertension, and hyperglycemia. Previous studies have shown that obesity is associated with progression of breast cancer, due to the augmented level of enzyme aromatase and increased production of estrogen caused by obesity [22, 23]. At the same time, patients with metabolic syndrome have a higher risk of cardiovascular disease. Obesity has been found to be a risk factor for cardiotoxicity of anthracyclines and trastuzumab in breast cancer patients [24]. Although we did not find a correlation of obesity with GLS or LVEF, further study with large scale of people is needed to be designed to confirm this correlation.

In addition, we compared difference of LVEF and GLS between the breast cancer patients with metabolic syndrome after cancer-related therapy and those without metabolic syndrome after therapy. We found that although there was no significant difference in LVEF between the two groups, the breast cancer patients with metabolic syndrome had a decrease of GLS, compared with those without metabolic syndrome, indicating that the breast cancer patients with metabolic syndrome were more prone to suffer from the subclinical myocardial dysfunction. The patients with metabolic syndrome were susceptible to the toxicity of cancer-related therapy, possibly due to many mechanisms. Firstly, patients with obesity and dyslipidemia often have myocardial steatosis, which could be a reason of deterioration of myocardium [25, 26]. Secondly, obesity and hypertension in patients with metabolic syndrome could lead to increased preload and after-load of the heart, resulting in the impairment of left ventricular function [27]. Thirdly, oxidative stress was increased in patients with metabolic syndrome, which may cause the heart to be more sensible to the toxicity of the cancer-related therapy [28]. Therefore, we should closely monitor the possible subclinical myocardial damage in breast cancer patients, especially those with metabolic syndrome, during cancer-related therapy.

There are several limitations of our studies. Firstly, our study is a single-center, cross-sectional study, although consecutive patients were enrolled in our study. Secondly, the number of the patients in our study is small; therefore, other factors may not be found by multivariate linear regression analysis. Thirdly, we investigated patients at different time points after completion of treatment, possibly resulting in a loss of data with change of GLS and LVEF. Therefore, further studies with large scale of people are needed.

## 5. Conclusions

We found that breast cancer patients with metabolic syndrome after cancer-related comprehensive treatment have a reduction of GLS and LVEF. GLS should be routinely performed to early identify subclinical myocardial damage of patients, in order to prevent the cardiotoxicity of cancer-related comprehensive therapy.

## Figures and Tables

**Table 1:** Baseline characteristics of breast cancer and noncancer patients with metabolic syndrome.

	Breast cancer patients with metabolic syndrome	Noncancer patients with metabolic syndrome	*P* value
Demographics			
Age (years)	49 ± 8	52 ± 10	0.442
BMI (kg/m^2^)	27.8 ± 3.2	27.3 ± 2.6	0.674
Waist circumference (cm)	92.6 ± 7.4	93.2 ± 8.3	0.542

Cardiovascular risk factors			
Coronary heart disease	0	0	—
Hypertension	9 (20%)	11 (24%)	0.342
High blood glucose	13 (29%)	15 (33%)	0.573
Dyslipidemia	26 (58%)	24 (53%)	0.483
Beta-blockers	2 (4%)	3 (7%)	0.231
ACE inhibitors	3 (7%)	2 (4%)	0.323

Vital sign			
Systolic blood pressure (mmHg)	122 ± 18	125 ± 15	0.673
Diastolic blood pressure (mmHg)	78 ± 10	76 ± 11	0.523

Cholesterol level (mmol/L)			
Total cholesterol	4.88 ± 0.97	5.02 ± 1.14	0.734
LDL-c	3.11 ± 0.79	3.15 ± 0.98	0.634
TG	1.82 ± 1.00	1.79 ± 1.15	0.667
Fasting glucose (mmol/L)	5.40 ± 0.97	5.56 ± 1.03	0.782

Breast cancer side			
Left	22 (49%)	—	—
Right	22 (49%)	—	—
Both	1 (2%)	—	—

Comprehensive therapy			
Chemotherapy	45 (100%)	—	—
Anthracycline use	31 (69%)	—	—
Trastuzumab use	20 (44%)	—	—
Both of anthracycline and trastuzumab	11 (24%)	—	—
Left-side radiotherapy	16 (36%)	—	—

Values as mean ± SD, or *n* (%). LDL-c: low density lipoprotein cholesterol; TG: triglyceride.

**Table 2:** Multivariate analysis for LVEF in breast cancer patients with metabolic syndrome after treatment.

Factor	*β*	SE	*P* value
Age	0.328	0.127	0.014
BMI	0.343	0.270	0.211
Anthracycline	−0.020	2.073	0.992
Trastuzumab	−1.442	1.901	0.453
Left-side radiotherapy	2.133	1.802	0.244

**Table 3:** Multivariate analysis for GLS in breast cancer patients with metabolic syndrome after treatment.

Factor	*β*	SE	*P* value
Age	0.014	0.076	0.855
BMI	−0.222	0.154	0.160
Anthracycline	0.767	1.194	0.525
Trastuzumab	2.489	1.107	0.031
Left-side radiotherapy	0.578	1.077	0.595

## Data Availability

The data used in the study can be provided upon request. This manuscript is available as a preprint on Research Square.

## Abbreviations

LVEF: Left ventricular ejection fraction
GLS: Global longitudinal strain
BNP: Type B natriuretic peptide
TnI: Troponin I.

## Supplementary Materials

Supplementary Materials Table S1: baseline characteristics of breast cancer patients with and without metabolic syndrome after therapy.
