# Supplementary material for: Identification of Subclinical Myocardial Dysfunction in Breast Cancer Patients with Metabolic Syndrome after Cancer-Related Comprehensive Therapy
Source: Cardiol Res Pract. 2021 Mar 2;2021:6640673. doi: 10.1155/2021/6640673 (PMC7943305; doi:10.1155/2021/6640673)
Supplement: Supplementary Materials — Table S1: baseline characteristics of breast cancer patients with and without metabolic syndrome after therapy. [file 6640673.f1.docx]

Supplementary Table 1 Baseline characteristics of breast cancer patients with and without metabolic syndrome after therapy

|  | Breast cancer patients with metabolic syndrome | Breast cancer patients without metabolic syndrome | P value |
| --- | --- | --- | --- |
| Demographics |  |  |  |
| Age (years) | 49±8 | 49±8 | 0.752 |
| BMI (kg/m^2^) | 27.8±3.2 | 22.8±1.0 | <0.001 |
| Cholesterol level (mmol/L) | | | |
| Total cholesterol | 4.88±0.97 | 4.2±0.38 | 0.039 |
| LDL-c | 3.11±0.79 | 2.6±0.57 | 0.042 |
| TG | 1.82±1.00 | 1.50±0.27 | 0.048 |
| Fasting glucose(mmol/L) | 5.40±0.97 | 5.39±0.44 | 0.952 |

Values as mean ± SD

LDL-c = low density lipoprotein cholesterol; TG = triglyceride
